# Two Children With Novel TRPC6 Spontaneous Missense Mutations and Atypical Phenotype: A Case Report and Literature Review

**DOI:** 10.3389/fped.2020.00269

**Published:** 2020-05-15

**Authors:** Meiqiu Wang, Ren Wang, Xu He, Min Yu, Zhengkun Xia, Chunlin Gao

**Affiliations:** ^1^Department of Pediatrics, Jinling Hospital, The First School of Clinical Medicine, Southern Medical University, Nanjing, China; ^2^Department of Pediatrics, Jinling Hospital, Nanjing Medical University, Nanjing, China; ^3^Department of Pediatrics, Jinling Hospital, Nanjing, China; ^4^Department of Neonatology, Taizhou People's Hospital, Taizhou, China

**Keywords:** TRPC6, SRNS, proteinuria, Genetic testing, immune complex-mediated glomerulonephritis

## Abstract

**Background:** The phenotypes of TRPC6 mutations have been reported mainly in familial and sporadic focal segmental glomerulosclerosis (FSGS), which can occur in both adults and children. Herein, we report on two children with novel TRPC6 spontaneous missense mutations associated with immune complex-mediated glomerulonephritis and minor glomerular abnormality (MGA) that showed to be resistant to corticosteroids and other immunosuppressants.

**Case Presentation:** A 9-year-old girl presented with steroid-resistant nephrotic syndrome (SRNS), while another 11-year-old boy developed proteinuria at 7 years old. Treatment with a variety of immunosuppressants had no effect, and the renal biopsy showed immune complex-mediated glomerulonephritis and MGA. No members of their family were clinically affected. Genetic testing was performed in the two patients, revealing two novel spontaneous missense mutations in TRPC6—N110S and P112R. The girl developed end-stage renal disease (ESRD) 5 months after onset while the boy continued to have sub-nephrotic range proteinuria and normal creatinine.

**Conclusions:** Two novel TRPC6 mutations were associated with the atypical phenotype—immune complex-mediated glomerulonephritis and MGA, rather than FSGS as previously reported. Their rates of disease progression are different. Genetic testing is helpful to identify the etiology and avoid the side effects brought on by immunosuppressants.

## Introduction

TRPC6, which encodes the protein transient receptor potential cation channel protein, is a non-selective cation channel, which is expressed in podocytes and interacts with nephrin and podocin to participate in signal transduction, cell polarization, skeletal structure stabilization, and other physiological functions between podocytes (1). In 2005, Winn identified an autosomal dominant family of focal segmental glomerulosclerosis (FSGS) with a point mutation in the TRPC6 gene on chromosome 11q (2). Since then, some new mutations of TRPC6 have been reported. It was confirmed that 24 mutations of the TRPC6 gene were involved in the pathogenesis of FSGS (Table 1). However, other pathological types associated with TRPC6 mutations have been less reported. Herein, we report on two children with novel TRPC6 mutations related to immune complex-mediated glomerulonephritis and minor glomerular abnormality (MGA).

**Table 1:** Mutations in TRPC6 protein currently identified to cause kidney disease.

**TRPC6 mutation**	**Published time**	**Effect on ion channel function**	**Ethnicity**	**Phenotype**	**Age at presentation (years)**	**Change in current amplitude**	**References**
P112Q	2005-07	Increased current amplitude	Caucasian	AD FSGS	30–40	Yes	(2)
N143S	2005-07	Not evaluated	African American	AD FSGS	30–40	None identified	(1)
	2009-11	Increased current amplitude	Caucasian	AD FSGS	27–39	Yes	(3)
S270T	2005-07	Not evaluated	Colombian	AD FSGS	17–52	None identified	(1)
K874X	2005-07	Not evaluated	Polish	AD FSGS	27–57	None identified	(1)
R895C	2005-07	Increased current amplitude	Mexican	AD FSGS	18–46	Yes	(1)
	2011-04	Not evaluated	Caucasian	AD collapsing FSGS	21–38	None identified	(4)
	2017-04	Not evaluated	Caucasian	AD FSGS	30	None identified	(5)
	2017-08	Not evaluated	Caucasian	AD FSGS	5	None identified	(6)
	2020	Not evaluated	Japanese	C1q nephropathy	3	None identified	(7)
E897K	2005-07	Increased current amplitude	Irish and German	AD FSGS	24–35	Yes	(1)
Q889K	2008-11	Increased current amplitude	Chinese	AD FSGS	35–41	Yes	(8)
	2013	Not evaluated	Chinese	FSGS	35–48	None identified	(9)
M132T	2009-11	Increased current amplitude and delayed channel inactivation	Caucasian	AD FSGS	9–30	Yes	(3)
	2010-02	Not evaluated	Caucasian	FSGS	8	None identified	(10)
G109S	2009-05	Probably damaging	Caucasian	FSGS	21	None identified	(11)
N125S	2009-05	Probably damaging	Caucasian	sporadic FSGS	41	None identified	(11)
	2011-06	Increased intracellular calcium	Caucasian	MCD	4	Yes	(12)
	2011-06	Increased intracellular calcium	Caucasian	IgAN with MPGN-like pattern	14	Yes	(12)
L780P	2009-05	Possibly damaging	Caucasian	sporadic FSGS	7	None identified	(11)
89fsX8	2010-02	Not evaluated	Caucasian	FSGS	7	None identified	(10)
G757D	2010-02	Not evaluated	Caucasian	FSGS	1	None identified	(10)
L395A	2011-04	Not evaluated	Turkish	sporadic FSGS	2.4	None identified	(13)
R360H	2011-05	Not evaluated	Caucasian	FSGS	34	None identified	(14)
H218L	2011-06	Increased intracellular calcium	Caucasian	sporadic FSGS	8	Yes	(12)
R895L	2011-06	Increased intracellular calcium	Caucasian	sporadic collapsing FSGS	1	Yes	(12)
D873fsX878	2013	Not evaluated	Korean	MCD	34	None identified	(15)
R175Q	2013-01	Increased intracellular calcium	Dutch	AD FSGS	27–53	Yes	(16)
A404V	2014-07	Not evaluated	Caucasian	MN	not mentioned	None identified	(17)
P15S	2014-07	Not evaluated	Caucasian	MN	not mentioned	None identified	(17)
R68W	2015	Increased intracellular calcium	Singaporean	FSGS	7–17	Yes	(18)
R175W	2017-02	Not evaluated	Chinese	FSGS	4 months	None identified	(19)
D130V	2018-11	Not evaluated	Iranian	FSGS	5	None identified	(20)
G162R	2018-11	Not evaluated	Iranian	FSGS	9	None identified	(20)
	2020	Not evaluated	Japanese	AD FSGS	2	None identified	(7)
G109D	2020	Not evaluated	Japanese	MGA	7	None identified	(7)
G875V	2020	Not evaluated	Japanese	AD FSGS	14	None identified	(7)
H145R	2020	Not evaluated	Japanese	AD FSGS	7	None identified	(7)

*MCD, minimal change disease; IgAN with MPGN-like pattern, IgA nephropathy with a membranoproliferative glomerulonephritis (MPGN)-like pattern; AD, autosomal dominant; FSGS, focal segmental glomerulosclerosis; MGA, minor glomerular abnormality; MN, membranous nephropathy*.

## Case Presentation

### Case 1

**Figure 1:**
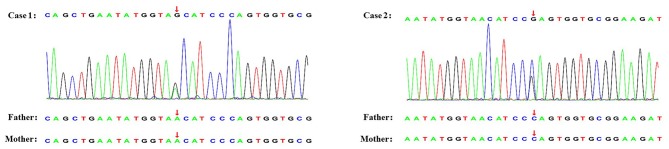
Genetic testing of two novel TRPC6 mutations. The arrow shows the position where base substitution results in changes in amino acids.

**Figure 2:**
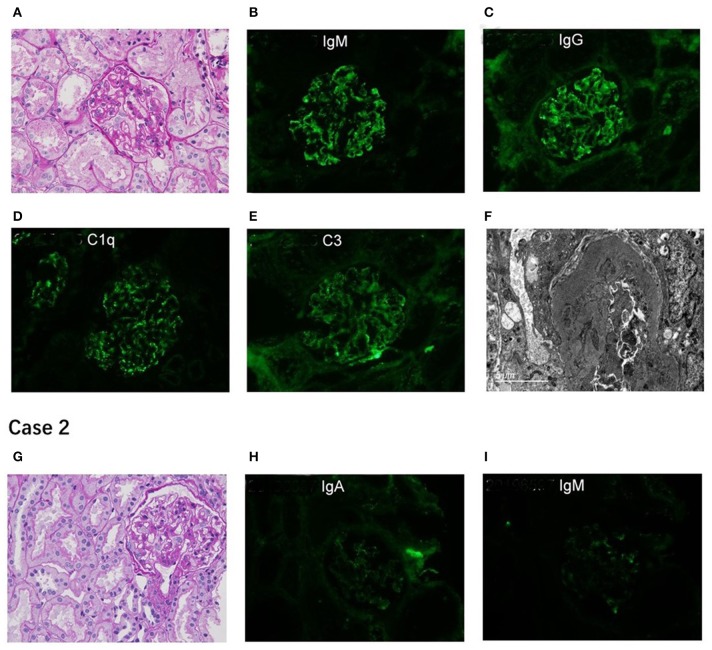
Renal pathological presentation of two proband. **(A,G)** for light microscopy (PAS×400); **(B–E,H,I)** for immunofluorescence; **(F)** for electron microscope. The figure of Case 2 is for subsequent biopsy.

A 10-year-old Chinese girl presented at the age of 9 years with steroid-resistant nephrotic syndrome (SRNS). The patient tested serologically negative for hepatitis B virus, human immunodeficiency virus, antinuclear antibodies (ANA) spectrum, and cytoplasmic and perinuclear antineutrophil cytoplasmic autoantibodies. She had periorbital edema and all other physical examinations were normal. She had a normal cardiac exam, normal nails and patellae, and no rashes or arthritis. Her percutaneous kidney biopsy demonstrated 6/26 showing as globally obsolete, 3/26 showing segmental sclerosis, and 1 having a cellular crescent. Mesangial cellularity was mildly increased, and tubules showed a patchy atrophy. Immunofluorescence staining showed that 1+ for C3 and C1q, IgG and IgM were 2+. Electron microscopy revealed that the distribution of medium electron density was observed in the mesangial region and subendothelial basement membrane. No electron density was found in the epithelium of the glomerular basement membrane. The podocyte processes were widely fused (Figure 2). Her treatment included atorvastatin 10 mg daily, and furosemide as needed, titrated to symptoms. Tacrolimus and cyclophosphamide had been added to steroids at different times, without success in achieving remission. No members of the family (parents and a sister) were clinically affected. To identify the pathogeny, the child and her family underwent the Whole Exome sequencing. High-throughput sequencing was performed with an Illumina NovaSeq 6000 series sequencer, and not less than 99% of the target sequence was sequenced. The detected mutations were checked in various databases (e.g., dbSNP, ExAC, ESP, OMIM, HGMD, ClinVar, etc.). It showed that a new missense change (c.329A>G) was identified in exon 2 causing an asparagine-to-serine substitution(N110S) within the first ankyrin repeat of the TRPC6 protein (Figure 1). At the onset of 5 months, the child developed chronic renal insufficiency and began hemodialysis and peritoneal dialysis.

### Case 2

A 7-year-old Chinese boy presented 2 days after the onset of eyelid swelling and tonsillitis. Urine dipstick tested 2+ positive for protein with no blood and 24-h protein excretion was 0.88 g. Initial serum concentrations were normal except for a slightly low albumin 3.3 g/dl and a slightly high total cholesterol 238 mg/dl. Other laboratory results showed that ANA, complement, antistreptolysin O, and serum creatinine were normal, with an initial biopsy showing IgA nephropathy (IgAN) (Lee II, M1E1S0T0). He was not edematous and did not have hypertension. He had normal nails and patellae, a normal cardiac exam, and no rashes or arthritis. His medications included losartan potassium 50 mg daily and fosinopril 10 mg daily. Cyclosporine A, mycophenolate mofetil had been added to steroids at different times, without success in achieving remission. At age 11, a subsequent biopsy was performed. Light microscopy demonstrated 1 out of 14 segmental sclerotic glomeruli with no other abnormalities. Immunofluorescence microscopy tested slightly positive for IgA and IgM. Staining for IgG, C3, and C1q was negative (Figure 2). Electron microscopy showed no glomerulus. The histopathologic diagnosis was MGA. He had no family history of kidney disease. Next, the Whole Exome sequencing was performed on the patient and his parents (case 2 used the same detection method as case 1). It showed that a new missense change (c.335C>G) was identified in exon 2 causing a proline-to-arginine substitution (P112R) within the first ankyrin repeat of the TRPC6 protein (Figure 1). He had been taking angiotensin-converting enzyme inhibitor (ACEI) as his main treatment. Current serum creatinine was 56 μmol/L, and 24-h urine showed proteinuria 1 g/day.

## Discussion

TRPC6 interacts with nephrin and podocin and is an important component of the podocyte slit diaphragm (3). Mutations in genes encoding the podocyte structural proteins lead to the development of proteinuria, bringing about progressive kidney failure. To date, a total of 28 novel TRPC6 mutations have been associated with FSGS, SRNS, and proteinuria (Table 1). Here we first identified two novel TRPC6 mutations (N110S, P112R) that manifest as diverse types of renal pathology with early-onset, multidrug resistance, and different clinical outcomes.

Winn identified an autosomal dominant family of FSGS with a point mutation in the gene TRPC6 (2). As shown in Table 1, we found that the incidence of TRPC6 mutation was more familial and less sporadic. The pathological type of these two children was not FSGS and the parents were not clinically affected. But the two children were resistant to immunosuppressants, which we suspected may be caused by gene spontaneous mutations. As a result, the two children tested negative for NPHS2, NPHS1, WT1, ACTN4, COL4A5, and CD2AP genes but positive for TRPC6. Their parents' genetic testing was normal. It shows the importance of environmental or other factors for mutations.

Several autoimmune diseases sporadically cause immune-complex deposition nephropathy. Circulating immune complexes because of autoimmune disease have been assumed to activate the classical complement pathway, which leads to glomerular injury (21). In case 1, the girl's response to treatment and laboratory tests would suggest that it was a non-immune-complex deposition disease. Previous studies have reported cases of IgAN and C1q nephropathy associated with TRPC6 mutations (7, 22), but there is no further explanation for the deposition of immune complexes in the kidney. It has been reported that several candidate genes relating to IgAN are involved in cytokine pathways, immunoregulation, and glycosylation (23, 24). Cox et al. identified 23 genes with candidate pathogenic variants functionally related to a large network of immune-related pathways. They speculated that the IgAN disease status may be affected by a series of mutations that affect immune-related networks (25). Milillo et al. identified the SPRY2 gene related to IgAN, which is part of the MAPK/ERK pathway (26). So, we hypothesized that variants in some pathways caused by TRPC6 mutations are risk factors for the development of immune-complex deposition nephropathy.

According to earlier studies, it has been identified that heterozygous mutations of TRPC6 cause late-onset autosomal-dominant FSGS. Heeringa et al. first reported that an M132T mutation in the TRPC6 gene can lead to early-onset FSGS in 2009 (3). Later, more and more TRPC6 mutations associated with kidney disease have been reported in children. The onset-age of the two children we reported are 7 and 9 years. Heeringa et al. believed that both a direct increase in calcium influx and an impaired channel inactivation may result in a more severe or earlier disease onset (3). It is shown in animal experiments that transient overexpression of wild-type TRPC6 in murine glomeruli causes rapid onset proteinuria (27).

The PolyPhen program predicted the TRPC6P112R(c.335C>G) mutation in case 2 to be “probably damaging” and the SIFT program anticipated this mutation to be “Deleterious” (Table 2). The mutation was not found in any public database of SNP. Winn indicated that Pro112 is highly conserved in TRPC protein homologs from multiple species and the peak intracellular concentrations were considerably higher in cells expressing TRPC6P112Q(c.335C>A) as compared with WT controls (2). The mutation in case 2 is also in Pro112, but it causes a proline-to-arginine substitution. We speculated that P112R may increase the current amplitude. The clinical manifestations and pathological type caused by P112R in our report are less severe than P112Q. The mechanism needs to be further explored. Additionally, other genetic and/or environmental factors may have an important impact on disease expressivity and penetrance. Two renal biopsies of case 2 had different outcomes, possibly because of immunofluorescence trapping. Nagano et al. found that 62% were FSGS and 28% were MGA in the histopathological diagnosis of 230 Japanese patients with proteinuria (7). MGA is a common pathological type of proteinuria, but only 1 case of MGA with normal renal function related to TRPC6 has been reported. MGA associated with TRPC6 mutation may progress slowly.

**Table 2:** Basic characteristics and clinical manifestations of 2 children.

**Patient number**	**Age (Years)**	**Biopsy**	**Phenotype**	**Age at diagnosis (Years)**	**Response to drugs (Steroids/CNI)**	**ESRD (Yes/No)/Years**	**Mutation**	**Protein change**	**The PolyPhen program**	**The SIFT program**	**Protein domain**
1	10	Immune complex-mediated glomerulonephritis	SRNS	9	No/No	Yes/9	c.329(exon2)A>G	p. N110S	Probably Damaging	Deleterious	ANK1
2	11	1. IgA nephropathy 2. minor glomerular abnormality	Proteinuria	7	No/No	No	c.335(exon2)C>G	p. P112R	Probably Damaging	Deleterious	ANK1

*SRNS, steroid-resistant nephrotic syndrome; CNI, calcineurin inhibitor; ESRD, End-Stage Renal Disease; ANK1, Ankyrin Repeat 1;1 for initial biopsy; 2 for subsequent biopsy*.

The PolyPhen program predicted the TRPC6N110S(c.329A>G) mutation in case 1 to be “probably damaging” and the SIFT program anticipated this mutation to be “Deleterious” (Table 2). The N110S mutation, which was not found in any public database of SNP, is located very close to P112Q, within the same ankyrin repeat. Considering their close location, we speculated that the channel kinetics of both mutants would be similar. The girl in case 1 entered end-stage renal disease (ESRD) only 5 months after the onset of the disease. One patient with IgAN associated with TRPC6 mutation had normal renal function (12), while another patient with C1q nephropathy had developed ESRD in the previous report (7). They have different rates of disease progression, and IgAN may progress more slowly. As it was a novel mutation that had not been previously reported on, and because we did not enter the molecular level to learn about the mechanism, the possible explanation we can offer is that the children may have proteinuria at an early stage, but it is not detected in time.

The girl in case 1 presented with SRNS and the boy in case 2 presented with proteinuria. After the failure of steroid therapy, they added immunosuppressants to relieve proteinuria. But it still did not work. A German study showed that 81% of patients with genetic SRNS did not respond to calcineurin inhibitor (CNI)—cyclosporin A (28). There are 28 TRPC6 mutations shown in Table 1; eleven (P112Q, N143S, R895C, E897K, Q889K, M132T, N125S, H218L, R895L, R175Q, R68W mutation) were gain-of-function mutations that resulted in increasing calcium channel activity (1–3, 8, 12, 16, 18). CNIs may affect the CaN-NFAT signaling pathway, which may promote cell apoptosis and destroy the podocyte actin cytoskeleton, thereby improving proteinuria (29). However, two of our patients were resistant to CNI. To avoid the side effects of immunosuppressants, they have stopped using the drugs. At present, the treatment goal in both patients is to protect kidney function, delay the progression of renal decline, and promote symptomatic management. Currently, the girl in case 1 receives regular peritoneal dialysis, while the boy in case 2 only takes ACEI orally.

Herein, we reported two new mutations in the TRCP6 gene that are related to different types of renal pathology, rather than FSGS as previously reported in the literature. But their diseases progress at different rates. Even though the PolyPhen and the SIFT program are very useful for predicting probable mutations, to date the functional assay is the definitive step to determine if a variant is a mutation. The lack of animal model experiments is a limitation of our research. We hypothesized that mutations in certain pathways caused by TRPC6 mutations may be responsible for the development of immune-complex deposition nephropathy in case 1. However, the exact mechanism of this disease needs to be confirmed by further experiments.

## Conclusions

Two novel TRPC6 mutations were associated with atypical phenotype—immune complex-mediated glomerulonephritis and MGA. Their rates of disease progression are different. Genetic testing is helpful to identify the etiology and avoid the side effects brought on by immunosuppressants.

## Ethics Statement

Written informed consent was obtained from the minor(s)' legal guardian/next of kin for the publication of any potentially identifiable images or data included in this article.

## Author Contributions

MW, RW, XH, MY, ZX, and CG drafted the manuscript or revised it critically for important intellectual content, provided final approval of the version to be published, and agreed to be accountable for all aspects of the work in ensuring that questions related to the accuracy or integrity of any part of the work were appropriately investigated and resolved.

## Conflict of Interest

The authors declare that the research was conducted in the absence of any commercial or financial relationships that could be construed as a potential conflict of interest.
